# Effects of acupuncture synchronized rehabilitation therapy on upper limb motor and sensory function after stroke: a study protocol for a single-center, 2 × 2 factorial design, randomized controlled trial

**DOI:** 10.3389/fneur.2023.1162168

**Published:** 2023-09-28

**Authors:** Zifu Yu, Xiaoxia Yang, Fang Qin, Tiantian Ma, Jie Zhang, Xiaoxuan Leng, Hongyan Bi, Xihua Liu

**Affiliations:** ^1^College of Rehabilitation Medicine, Shandong University of Traditional Chinese Medicine, Jinan, China; ^2^School of Nursing, Shandong University of Traditional Chinese Medicine, Jinan, China; ^3^The First Clinical Medical College of Shandong University of Traditional Chinese Medicine, Jinan, China; ^4^Department of Rehabilitation, Affiliated Hospital of Shandong University of Traditional Chinese Medicine, Jinan, China

**Keywords:** stroke, upper limb, acupuncture synchronized rehabilitation therapy, motor function, sensory function, activities of daily living, factorial design, randomized controlled trial

## Abstract

**Background:**

Upper limb function reconstruction has been an important issue in the field of stroke rehabilitation. Due to the complexity of upper extremity dysfunction in stroke patients, the clinical efficacy produced by central or peripheral stimulation alone is limited. For this reason, our group has proposed acupuncture synchronized rehabilitation therapy (ASRT), i.e., simultaneous scalp acupuncture and intradermal acupuncture during rehabilitation. Pre-experiments results showed that this therapy can effectively improve the motor and sensory functions of upper limbs in post-stroke patients, but the clinical efficacy and safety of ASRT need to be further verified, and whether there is a synergistic effect between scalp acupuncture and intradermal acupuncture also needs to be studied in depth. Therefore, we designed a randomized controlled trial to compare the efficacy and safety of different therapies to explore a more scientific “synchronous treatment model.”

**Methods:**

This is a single-center, randomized controlled trial using a 2 × 2 factorial design. We will recruit 136 stroke survivors with upper extremity dysfunction and randomize them into four groups (*n* = 34). All subjects will undergo routine treatment, based on which the Experimental Group 1: rehabilitation training synchronized with intradermal acupuncture treatment of the affected upper limb; Experimental Group 2: rehabilitation training of the affected upper limb synchronized with focal-side scalp acupuncture treatment, and Experimental Group 3: rehabilitation training synchronized with intradermal acupuncture treatment of the affected upper limb synchronized with focal-side scalp acupuncture treatment; Control Group: rehabilitation training of the affected upper limb only. The intervention will last for 4 weeks, 5 times a week. Both acupuncture treatments will be performed according to the Revised Standards for Reporting Interventions in Clinical Trials of Acupuncture (STRICTA). The primary outcome indicators for this trial are Fugl-Meyer Assessment-Upper Extremity and Somatosensory Evoked Potential. Secondary outcome indicators include Wolf Motor Function Test, Upper Extremity Function Test, revised Nottingham Sensory Assessment Scale, Diffusion Tensor Imaging, and Modified Barthel Index. The incidence of adverse events will be used as the indicator of safety.

**Discussion:**

The study will provide high-quality clinical evidence on whether ASRT improves upper limb motor and sensory function and activities of daily living (ADL) in stroke patients, and determine whether scalp acupuncture and intradermal acupuncture have synergistic effects.

**Clinical trial registration:**

https://www.chictr.org.cn/, Chinese Clinical Trial Registry [ChiCTR2200066646].

## 1. Introduction

Stroke, also known as cerebrovascular accident, is currently the second leading cause of disability worldwide and is characterized by high morbidity and disability rate (1, 2). Thanks to continuous advances in various diagnoses and treatment techniques, the mortality rate of stroke patients has decreased in recent years (3). However, increased survival of stroke patients has also heralded an increase in chronic stroke patients (4), and most surviving stroke patients tend to have varying degrees of dysfunction left over (5). The lack of function obviously reduces their quality of life and imposes a considerable burden on their families and society.

Studies have shown that 65% of patients have residual upper Extremity dysfunction 6 months after stroke occurred (6). The function of upper limb is closely related to ADL, and that persistent non-recovery of the hand and upper limb function is a major impediment to returning to home and work (7). In addition, the upper extremity function is more delicate and complex, and involves more central parts of the brain, making it more difficult to recover (8–10). Therefore, the reconstruction of upper limb function has been an important subject in the field of stroke rehabilitation.

In recent years, several experiments have pointed out that rehabilitation during scalp acupuncture with needle retention is more effective than rehabilitation after scalp acupuncture in enhancing the upper extremity function of stroke patients, suggesting that the effects of both may not simply be additive (11, 12). To further investigate this issue, our research team conducted a systematic evaluation using Meta-analysis, and the results showed that the simultaneous treatment improved the FMA and MBI scores of stroke patients significantly compared to conventional acupuncture combined with rehabilitation and that this “synchronized treatment mode” better mobilized the neurofeedback mechanism, and thus help patients to establish normal motor patterns and ADL ability (13).

Currently, “simultaneous treatment modalities” of acupuncture and rehabilitation are commonly used in clinical practice including intradermal acupuncture synchronized rehabilitation and scalp acupuncture synchronized rehabilitation, both of which have been clinically verified (14, 15). Intradermal acupuncture stimulates peripheral acupoints to transmit nociceptive sensation to the brain and produce motor response after information integration, thus improving patients' movement function (16); while scalp acupuncture can strengthen sensory integration by directly stimulating local brain areas, thus regulating motor function (17). However, upper limb dysfunction after stroke is extremely complex, and the clinical efficacy produced by considering local stimulation such as central or peripheral alone is limited. Previous research has shown that there is a strong connection between neuroplasticity theory and Traditional Chinese Medicine (TCM) theory (18). Therefore, our research team applied the holistic concept of TCM to stroke rehabilitation, and after reviewing a large amount of literature and based on our previous clinical practice, then proposed Acupuncture Synchronized Rehabilitation Therapy (ASRT), which is one of the “simultaneous treatment modalities,” featuring both head and limb acupuncture, i.e., simultaneous scalp acupuncture and intradermal acupuncture during rehabilitation training, to maximize the activation of nerve conduction pathways, promote neurological remodeling and improve patient function. However, the clinical efficacy and safety of this therapy need to be further verified. In addition, whether there are synergistic effects between the head needle and the intradermal needle need to be further investigated.

Given this, we designed this clinical randomized controlled trial to compare the efficacy and safety of upper limb rehabilitation training with intradermal acupuncture therapy or scalp acupuncture therapy or all three at the same time (i.e., ASRT) and upper limb rehabilitation training alone, to explore a more scientific and rational treatment model, so as to establish a new and efficient stroke rehabilitation program. In this study, we will establish a scientific basis for the rehabilitation of stroke-related upper limb dysfunction.

## 2. Methods

### 2.1. Study design

**Figure 1:**
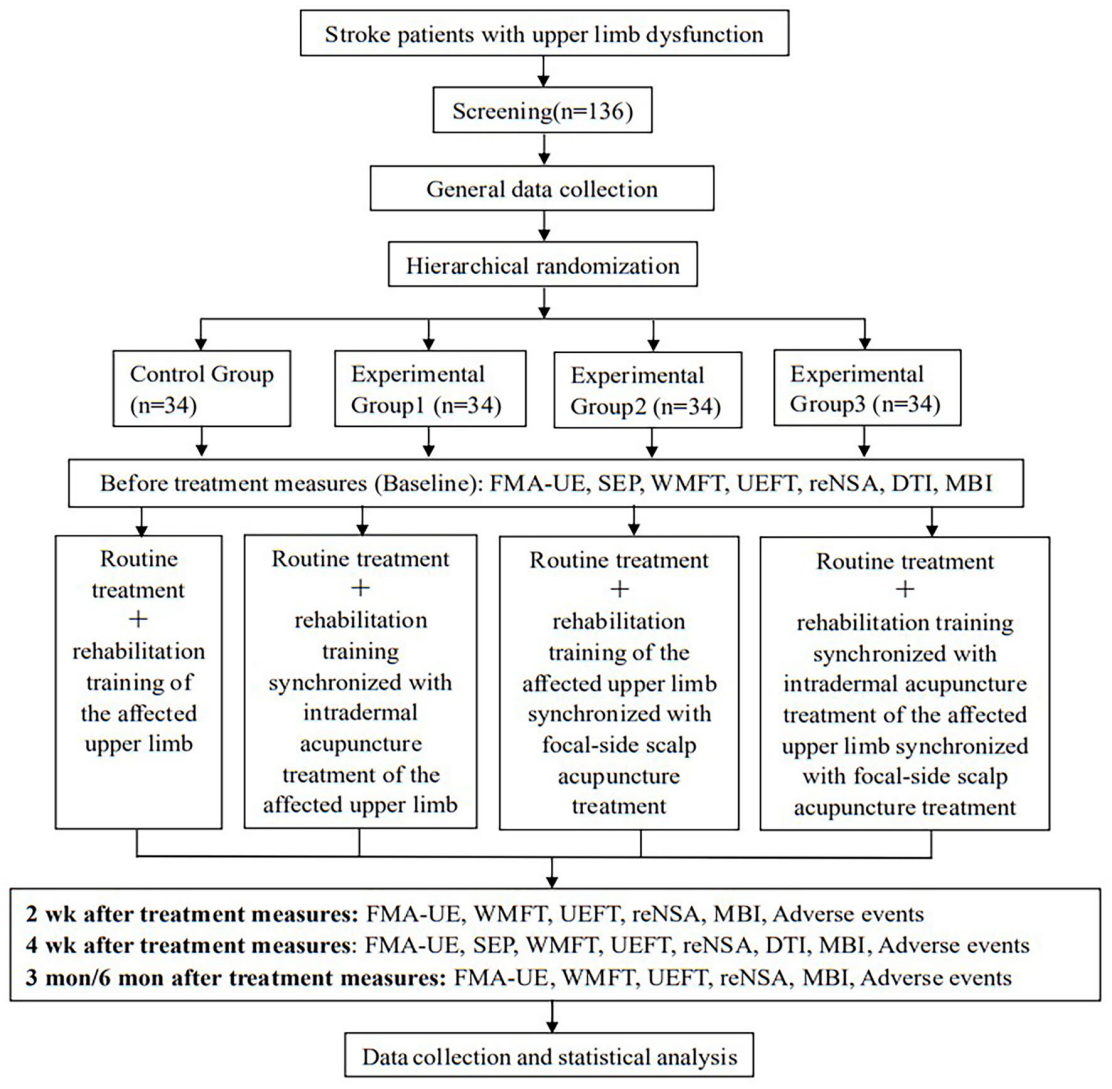
Trial flow chart.

This is a prospective, open, single-center, randomized controlled trial using a 2 × 2 factorial design. The two trial factors are the head needle and intradermal needle, each with two levels (with and without). Both intradermal acupuncture and scalp acupuncture are designed on the basis of the standard Revised Standards for Reporting Interventions in Clinical Trials of Acupuncture (STRICTA) (19). The study will include 136 stroke patients with upper limb dysfunction, grouped into four groups of 34. All subjects will undergo routine treatment, based on which the Experimental Group 1 (EG1): rehabilitation training synchronized with intradermal acupuncture treatment of the affected upper limb; Experimental Group 2 (EG2): rehabilitation training of the affected upper limb synchronized with focal-side scalp acupuncture treatment, and Experimental Group 3 (EG3): rehabilitation training synchronized with intradermal acupuncture treatment of the affected upper limb synchronized with focal-side scalp acupuncture treatment; Control Group (CG): rehabilitation training of the affected upper limb only. Head and intradermal needling will be performed in accordance with the Chinese national standard GB/T 21709.2-2021 (20) and the fifth edition of Science of Acupuncture and Moxibustion (21). Outcomes will be measured at baseline and a total of five time points at 2 weeks, 4 weeks, 3 months, and 6 months after treatment. Description of the protocol is shown in Table 1, and the study flow chart is presented in Figure 1.

**Table 1:** SPIRIT figure.

	**Study period**						
	**Screening**	**Baseline**	**Treatment**			**Follow up**	
**Time point**	**-t**_1_	**0**	**t**_1_	**t**_2_	**t**_3_	**t**_4_	**t**_5_
**Enrollment**							
Eligibility screen	√						
Informed consent	√						
Demographic information	√						
Medical history	√						
Random allocation		√					
**Interventions**							
Experimental group 1			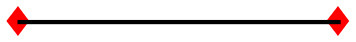				
Experimental group 2			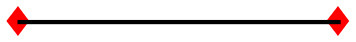				
Experimental group 3			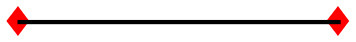				
Control group			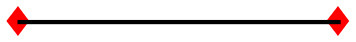				
**Assessments**							
FMA-UE		√		√	√	√	√
SEP		√			√		
WMFT		√		√	√	√	√
UEFT		√		√	√	√	√
reNSA		√		√	√	√	√
DTI		√			√		
MBI		√		√	√	√	√
Adverse events			√	√	√	√	√

-t_1_, patient screening; 0, baseline evaluation; t_1_, time of the start of treatment; t_2_, 2 weeks after treatment; t_3_, 4 weeks after treatment; t_4_, 3 months after treatment; t_5_, 6 months after treatment; FMA-UE, Fugl-Meyer Assessment-Upper Extremity; SEP, Somatosensory Evoked Potential; WMFT, Wolf Motor Function Test; UEFT, Upper Extremity Function Test; reNSA, revised Nottingham Sensory Assessment scale; DTI, Diffusion Tensor Imaging; MBI, Modified Barthel Index.

This study protocol was approved by the Ethics Committee of the Affiliated Hospital of Shandong University of Traditional Chinese Medicine (approval No. 2022-080-KY) on August 5, 2022, and this trial was registered with the Chinese Clinical Trial Registry (registration No: ChiCTR2200066646; date: December 12, 2022). This trial will be conducted in accordance with the Standard Protocol Items: Recommendations for Intervention Trials (SPIRIT) checklist (22).

### 2.2. Participant recruitment

The subjects will be hospitalized in the Rehabilitation Department of the Affiliated Hospital of Shandong University of Traditional Chinese Medicine. This department focuses on improving various functional impairments that occur after stroke through a combination of Chinese and Western medicine rehabilitation. Patients will be recruited by distributing brochures in the hospital, posting notices on bulletin boards, and publishing information on the WeChat public platform. Stroke patients who pass the eligibility screening will become subjects of this study. The research team will provide professional treatment for the subjects, and save the cost of instrument examination and scale evaluation. However, medication, rehabilitation, and acupuncture treatments are not free of charge. Patients interested in participating in this trial can contact the researchers by telephone or WeChat. Participation in the trial is voluntary and patients will be encouraged to ask the researcher as many questions as possible before they decide to participate until they fully understand the content, risks, and benefits of the trial. Details such as gender, age, duration of illness, type of stroke, side of hemiparesis, Brunnstrom stage, and Mini-mental state examination (MMSE) score will be recorded by the investigator on their respective Case Report Forms (CRFs) at the time of admission. During treatment, the researchers will collect and monitor the patient's scale assessment report, imaging, and electrophysiological data.

#### 2.2.1. Inclusion criteria

(1) Diagnosis by cranial CT and MRI and meeting the diagnostic criteria for stroke in Chinese Diagnostic Points for Various Types of Cerebrovascular Diseases 2019 (23); (2) The type of stroke is cerebral hemorrhage or cerebral infarction, all of which were unilateral limb hemiparesis with the first onset within 12 months; (3) The duration of the disease was 2 weeks-6 months after onset, with Brunnstrom stage II-IV of the upper limb; (4) Stable vital signs and no further progression of neurological symptoms; (5) Clear consciousness, MMSE > 17 points; (6) Patients aged 18–70 years, of any gender, without serious cardiac, cerebral, or renal complications; (7) Fully informed and voluntary participation in the study, cooperation with assessment and treatment, and signed informed consent form.

Patients who fulfill all of the conditions listed above will be considered for inclusion.

#### 2.2.2. Exclusion criteria

(1) Spatial neglect and severe visual, hearing, intellectual, and attention deficits; (2) Severe upper extremity spasticity with a modified Ashworth scale grading of ≥3; (3) Patients with a previous severe neurological disease or history of psychiatric disorders; (4) Presence of musculoskeletal joint disorders (e.g., fractures, arthritis, etc.) or neurological disorders (e.g., tremor, peripheral nerve injury, etc.) that affect the motor or sensory function of the affected upper extremity; (5) Other reasons for inability to cooperate with training or poor compliance; (6) Those who cannot tolerate head or intradermal needle treatment; (7) Unable to complete DTI or SEP testing; (8) Patients who have received relevant treatment may affect the efficacy observation index.

Patients with any of the above conditions will be excluded.

#### 2.2.3. Withdrawal criteria

(1) Patient-initiated withdrawal; (2) Patients who have certain complications or comorbidities during the trial and are not suitable to continue the trial; (3) The patient's condition deteriorates or another stroke occurs and a dangerous event may occur by continuing the trial.

#### 2.2.4. Termination criteria

The principal investigator has the right to cease the study at any time for reasons including, but not limited to, that continuing the study may harm the rights and interests of some subjects.

### 2.3. Randomization, allocation concealment, and blinding

In this study, a stratified blocked randomization method will be used to stratify patients by age (18–45 years old, 46–75 years old) and type of stroke (cerebral hemorrhage, cerebral ischemia), and SPSS 26.0 software (IBM, Armonk, NY, USA) will be used to stratify and randomize 136 patients who met the criteria for upper limb dysfunction after stroke. The patients will be randomly divided into EG1, EG2, EG3, and CG in a ratio of 1:1:1:1, with 34 patients in each group. The personnel responsible for randomization will not be involved in the process of patient recruitment, treatment, assessment, data collection, and statistical analysis. The grouping results will be placed in sealed, opaque envelopes. Subjects will be numbered according to the time of enrollment, and the appropriate envelopes will be given to the person performing the study intervention according to the number.

To exclude as much bias as possible, the assessors and statistical analysts will be blinded, with 2 assessors who are professionally and uniformly trained and 2 other staff members who are responsible for entering and analyzing the data, regardless of patient assignment and without contact with treatment staff.

### 2.4. Sample size

This study is a RCT with a 2×2 factorial design in which the FMA-UE scores of stroke patients were used as the primary outcome indicators. Based on the results of the preliminary pre-experiment and combined with the relevant literature (24), we used PASS15.0 software (PASS, Kaysville, UT, USA) for sample size estimation. Based on previous studies, we were informed that the mean values of FMA-UE scores after treatment for the four groups were 41.36, 48.21, 52.87, and 40.54, respectively. After calculation, the standard deviation of the two-factor interaction was 0.96, and the estimate of the overall standard deviation was 3.38. The test level was set at α = 0.05 (bilateral test) with a certainty of 1-β = 0.9. The sample size required was 34 cases in each group, and a total of at least 136 patients needed to be included, according to 1:1:1:1 parallel grouping, taking into account the 10% dropout rate of the study population.

### 2.5. Intervention

**Figure 2:**
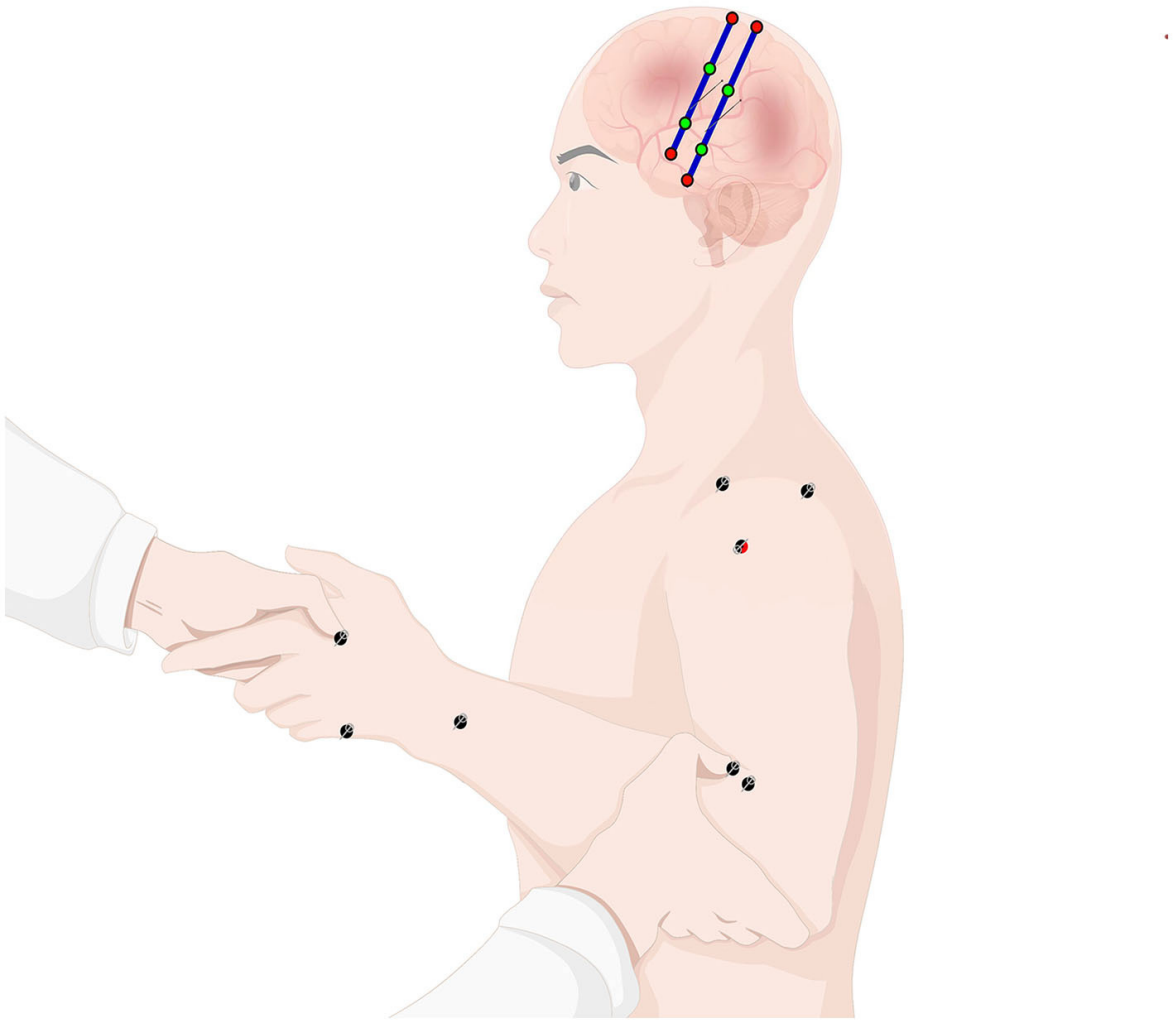
Schematic diagram of ASRT for upper limb dysfunction in stroke patients (this figure is partially drawn by Figdraw. ID: AYIWY42f22).

Based on conventional treatment, EG1: rehabilitation training synchronized with intradermal acupuncture treatment of the affected upper limb; EG2: rehabilitation training of the affected upper limb synchronized with focal-side scalp acupuncture treatment; EG3: rehabilitation training synchronized with intradermal acupuncture treatment of the affected upper limb synchronized with focal-side scalp acupuncture treatment, i.e., ASRT, the schematic diagram of which is shown in Figure 2, and CG: rehabilitation training of the affected upper limb. Subjects in each group will complete treatment for a fixed period of time, 30 min/session, 1 session/day, with 2 days off after treatment on 5 consecutive days per week for a total of 4 weeks.

#### 2.5.1. Conventional treatment

All patients will receive conventional treatment, including pharmacological treatment and rehabilitation. Pharmacological treatment applies anticoagulation, lipid regulation, blood pressure and blood glucose control, cerebral nerve nutrition, and improvement of cerebral metabolism to control complications and reduce functional impairment (25). Rehabilitation therapy includes movement therapy of the affected lower limb and trunk (neurodevelopmental therapy based on the new Bobath technique, proprioceptive neuromuscular facilitation technique (PNF), nerve stimulant therapy, balance function training, and gait training), physical factor therapy, occupational therapy (mainly ADL training), and speech-swallowing training. The above treatments are individualized and targeted, and appropriate interventions should be selected according to the degree and type of functional impairment of the patient. 5 days per week, for 4 weeks.

#### 2.5.2. Rehabilitation training of the affected upper limb

Individualized functional training will be used for the affected upper limb, which mainly included stretching training (continuous passive distraction to inhibit persistent contraction of spastic muscles), and new Bobath technique (control of key points to inhibit abnormal postural and motor patterns), and the PNF technique.

#### 2.5.3. Intradermal acupuncture treatment of the affected upper limb

*Acupuncture points selection:* Jianyu (LI15), Jianliao (TE14), Jiquan (HT1), Chize (LU5), Quze (PC3), Hegu (LI4), Houxi (SI3), Waiguan (TE5) points on the affected upper limb.

*Acupuncture method:* After sterilization of the acupoints, the granular intradermal needles (0.22 mm × 5 mm, Huanqiu, Suzhou, China.) will be selected, and the skin around the acupoints will be stretched to the sides with one hand, and the needle will be inserted into the acupuncture point subcutaneously by holding the needle handle with forceps in the other hand, then the needle handle will be covered with medical tape to hold the needle in place. The intradermal acupuncture will retain for 30 min, and during the period of needle retention, pressure will be performed every 5 min for 1 min each time.

*Acupuncture removal method:* The physician fixes the skin on both sides of the needle with one hand, removes the adhesive tape with the other hand, and then holds the needle handle with forceps and removes the needle.

#### 2.5.4. Scalp acupuncture treatment of the focal-side head

*Acupuncture points selection:* The middle part of 2/5 of the Anterior Oblique Line of Vertex-Temporal (MS6), from Qianding (DU21) oblique to Xuanli (GB6), and the middle part of 2/5 of the Posterior Oblique Line of Vertex-Temporal (MS7), from Baihui (DU20) obliquely to Qubin (GB7) on the side of the lesion.

*Acupuncture method:* Following routine local disinfection, a disposable stainless steel needle (0.35 mm × 40 mm, Huatuo, Suzhou, China) will be used, and the needle made an angle of 30° with the scalp and will be rapidly stabbed into the subscalp when the tip reaches the epicranial aponeurosis, the needle will be made parallel to the scalp and stabbed 25~35 mm, then the twisting technique will be performed rapidly and continuously, and the twisting speed will be kept at about 150 times per min for 2 min to be the patient to get Deqi sensation (26). The needle will be retained for 30 min, and the twisting technique will be performed every 10 min during the needle retention period.

*Acupuncture removal method:* the physician first slowly out the head needle to the subcutaneous, and then quickly pulls out, pulling the needle with a sterile cotton ball to press the needle hole to prevent bleeding.

### 2.6. Outcome measures

#### 2.6.1. Primary outcome measures

(1) *FMA-UE* will be used to evaluate the patients' upper limb movement function.

This scale is one of the most widely used clinical scales for assessing upper extremity motor recovery after stroke, with 33 items, divided into 3 levels of scores, 0, 1, and 2 points respectively, according to the degree of task completion, and the total score was 66, with higher patient scores suggesting better motor function. FMA-UE is quantified by indicators and has the advantages of being detailed, reliable, and sensitive, which has been established as a standardized assessment tool to measure upper limb movement function (27).

Assessment time points: baseline, and 2 weeks, 4 weeks, 3 months, and 6 months after treatment.

(2) *SEP* will be used to assess the sensory function.

SEP is the potential induced in different parts of the central nervous conduction pathway after the skin or peripheral nerve receives certain pulse electrical stimulation. It has the advantages of high sensitivity, strong specificity, and repeatability (28). As an objective test, it excludes the influence of patient's subjective factors, which makes the evaluation results more reliable; SEP not only provides an objective assessment of stroke limb sensory function and feedback on the therapeutic effect of limb rehabilitation, but also guides the formulation of limb rehabilitation protocols and clinical treatment, and is considered an important reference indicator for predicting the prognosis of stroke limb function (29, 30). The main observations of SEP are latency and wave amplitude, with latency related to the brain white matter and wave amplitude related to axons and cortex. N20 arises in the primary somatosensory cortex and is the first near-field potential of SEP (31). In this study, an EMG-evoked potential device (Dantec, Denmark) will be used for median nerve SEP testing. A saddle stimulating electrode will be placed 2–3 cm above the transverse carpal tunnel of the upper limb on the hemiplegic side of the patient. According to the 10–20 international system of EEG electrode placement (32), the recording electrodes were located at C3' & C4' of the head contralateral to the limb under test, and the reference electrode is placed at Fz. The electrode impedance will be kept below 5 kΩ. Constant current square wave pulses will be used with a frequency set at 5 Hz and a pulse width of 0.2 ms, with a stimulation intensity sufficient to elicit slight thumb movement. Two median nerve N20 SEP examinations will be performed for each evaluation, each time superimposed 200 times, and the latency and wave amplitude of the N20 wave will be recorded, and the average value of the two times will be taken as the final test result.

Assessment time points: baseline, and 4 weeks after treatment.

#### 2.6.2. Secondary outcome measures

(1) *WMFT* will be utilized to evaluate upper extremity motor function, the WMFT scale contains 15 assessment tasks, items 1–7 mainly test single-joint motor function, and items 8–15 test the ability of multiple joints to move. Each item is divided into 6 levels of scoring according to the degree of task completion, with a maximum score of 5 and a minimum score of 0. The total score is 75. Higher scores tend to indicate better upper limb motor function. This scale not only evaluates the quality of the patient's task completion but also provides a record of the time of task completion. It has been demonstrated to be highly reliable and valid for assessing upper extremity motor dysfunction after stroke (33).

Assessment time points: baseline, and 2 weeks, 4 weeks, 3 months, and 6 months after treatment.

(2) *UEFT* will also be used as a scale to evaluate upper limb motor function in this trial. This scale contains 33 activities, which are divided into categories I~VI, of which categories I ~IV mainly check the grasping and finger-pinching functions of the hand and categories V~VI mainly assess the overall function and coordination of the upper limbs. Each activity was divided into four levels, with a score of 0 indicating that all activities could not be completed; a score of 1 indicating that only part of the activities could be completed; a score of 2 indicating that the activities could be completed, but the movements were slow or clumsy; and a score of 3 indicating that the activities could be completed normally (34).

Assessment time points: baseline, and 2 weeks, 4 weeks, 3 months, and 6 months after treatment.

(3) *The Chinese version of reNSA* will be used to assess upper limb sensory function, which includes the sensory of light touch, temperature, pinprick, pressure, and proprioception in the shoulder, elbow, wrist, and hands, and stereognosis in both hands. Light touch, temperature, pinprick, and pressure sensation were divided into 3 levels, for a total of 64 points; proprioception was divided into 4 levels (0–3 points), for a total of 24 points. Stereognosis was assessed by asking patients to perceive different objects placed in their hands, a total of 10 points. Yang et al. conducted cross-cultural adjustment and reliability and validity tests of the scale and noted that the Chinese version of reNSA can be used to assess the sensory function of stroke patients and provide an objective basis for rehabilitation planning (35).

Assessment time points: baseline, and 2 weeks, 4 weeks, 3 months, and 6 months after treatment.

(4) *DTI* will be used to predict upper extremity movement function, which is a non-invasive magnetic resonance imaging technique that effectively observes and tracks the alignment and direction of white matter fiber tracts and the coherence and integrity of myelin sheaths by quantitatively assessing the differences in diffusion information of water molecule in brain (36), and can acutely track the changes in white matter microstructure of the brain (37). Studies have shown that injury of the posterior limb of the internal capsule (PLIC) is significantly associated with the prognosis of upper limb motor function (38). In this study, the Fractional anisotropy (FA) of the corticospinal tract of the PLIC will be measured on the focal side and the healthy side of the patient, and the ratio of FA (rFA) and FA asymmetry (FAasy) will be calculated.

Assessment time points: baseline, and 4 weeks after treatment.

(5) *MBI* will be used to assess patients' ADL. MBI can be used to predict the treatment effect and prognosis of patients. The scale contains 10 assessment tasks, including toileting, bathing, eating, grooming, etc. The total score is 100. Each task is categorized into 5 levels according to the patient's dependence, and each task and level has its corresponding score. The higher the patient's score, the lower the level of dependency, i.e., the greater the ability to live independently (39).

Assessment time points: baseline, and 2 weeks, 4 weeks, 3 months, and 6 months after treatment.

#### 2.6.3. Safety indicators

Incidence of adverse events = (number of patients with adverse events/total number of patients) × 100%.

*Assessment time points:* time of the start of treatment, 2 weeks, 4 weeks, 3 months, and 6 months after treatment.

#### 2.6.4. Adverse events

##### 2.6.4.1. Identification and judgment of adverse events

Adverse events can be any accident, signs, symptoms, diseases, or abnormal test results. These events should be considered adverse events as long as they occur after the intervention, whether or not they are related to the treatment.

*Adverse events of rehabilitation training:* Muscle stretching training may lead to local tissue discomfort, such as pain and swelling, and may even result in fractures in elderly patients with osteoporosis. Postural hypotension or even falls may result when performing postural transfer training. The rehabilitation therapists participating in the study are uniformly trained and skilled and will monitor the patient's blood pressure and heart rate when necessary.

*Adverse events of acupuncture treatment:* Pain, stagnant needles, bent needles, and even infection may occur. The needles should be inserted into the skin quickly to relieve pain. If stagnant needles occur, the needle retention time should be extended appropriately, and the patient should be instructed to relax and massage gently around the needle. If the needle handle is slightly bent, the needle should be slowly lifted out, and if the bending angle is too large, the needle should be lifted out in the direction of the bend. To prevent infection, acupuncture points should be sterilized before acupuncture, and the needle holes should be kept clean after needle removal. The acupuncturists have undergone extensive clinical practice and are able to ensure the safety of acupuncture treatment.

##### 2.6.4.2. Treatment of adverse events

The investigator will determine before each intervention whether the patient is fit to continue treatment and inform the patient of any discomfort during treatment with prompt feedback. Once the patient indicates discomfort, the researcher will immediately stop treatment and assess the patient's signs, symptoms, type, and degree of discomfort, and then decide what to do next.

##### 2.6.4.3. Recording, reporting, and management of adverse events

The researcher will use CRFs to record the adverse events that occurred during the study. If subjects withdraw from the trial due to adverse events, researchers will follow up until the adverse event is resolved. Any adverse events will be reported to the Ethics Committee.

### 2.7. Data collection, management, and monitoring

#### 2.7.1. Data collection

The original data (scale or device assessment results, etc.) of the subjects will be fully collected and recorded in the CRFs in a true, clear, complete and standardized manner. All items in the CRFs will be filled in, and if any content needs to be changed, it should be underlined and the time and reason for the change should be indicated, in addition to a signature next to it.

#### 2.7.2. Data management

All data (documents, evaluation reports, clinical trial results, etc.) will be stored in order in the hospital's archive, where they can be retrieved quickly. These files will be managed by the administrator, anyone can only enter the archives with permission. In addition, paper documents will be scanned and stored encrypted in the computer.

#### 2.7.3. Data monitoring

The inspector will monitor whether the experiments are conducted in accordance with the research protocol and compliance with the relevant regulations, whether there are errors or omissions in the raw data, whether all CRFs are complete and accurate, and whether they deviate from the raw data.

### 2.8. Statistical analysis methods

#### 2.8.1. Data description

The results of the trial will be analyzed according to the intention-to-treat principle. The researchers will collect the follow-up data at each time point, create a database with information and each observed index data of the four groups of cases, and use SPSS26.0 software for statistical analysis. The Shapiro-Wilk method will be used to test the normality of the measurement data, which will be expressed as the mean ± standard deviation (SD) for normal distribution; For non-normally distribution, *M* (*P*_25_*, P*_75_) will be used, and the count data were statistically described using the number of cases and percentages.

#### 2.8.2. Analysis methods

The major analysis will be based on the results of the assessment of 4 weeks after treatment. FMA-UE score, SEP-related index, UEFT score, WMFT score, reNSA score, MBI score, and DTI-related data are all measurement data. For within-group comparisons, if the data conformed to normal distribution, paired *t*-test will be used to compare SEP and DTI results, and other data will be performed using repeated measures ANOVA, and least significant different (LSD) methods will be used for pair comparison; for data that do not fit the normal distribution, the Wilcoxon rank-sum test will be used for SEP and DTI results, and the Generalized estimating equation (GEE) approach will be used for all other data. Between-group comparisons will be performed by analysis of variance with a factorial design, and interaction effects and main effects will be analyzed. In addition, Pearson correlation analysis or Spearman's analysis will be used to analyze the correlation among indicators. For the incidence of adverse events among groups, Fisher's exact test will be used. Statistical treatments were all performed using a two-sided test with an inspection level of α = 0.05.

### 2.9. Quality control

All interventions and assessment methods will be completed by trained acupuncturists and rehabilitation therapists.

According to the relevant legal regulations in China, if an adverse event occurs during the trial, the subject group will bear the corresponding subsequent medical expenses of the subject. If patients need to be hospitalized due to a serious adverse event, the research group will provide financial compensation for nutritional expenses, lost wages, etc.

### 2.10. Ethics and dissemination

This study protocol met the requirements of the Declaration of Helsinki (2013 version) (40). All subjects will be enrolled voluntarily in this trial and will be fully informed of the trial protocol prior to the start of the clinical trial. Subjects who ultimately decide to participate in the study will be required to sign a written informed consent form.

The investigator is obliged to submit periodic interim trial reports in accordance with the relevant requirements of the Institutional Review Board. Upon completion of the trial, the investigator will report to the Ethics Committee that the trial has been completed. If the study protocol is not fully followed, i.e., there is a deviation, the investigator will fill in the protocol violation record and report it to the ethics committee in a timely manner. No modifications to the study protocol will be made by anyone other than the research designer. Any changes to the study protocol will be made by way of revision and submitted to the Ethics Committee for approval, and the revision will ultimately be completed at the Chinese Clinical Trial Registry.

The results of this study should be published only after obtaining the consent of the principal investigator. During the study period, all information of the participants will be kept confidential in strict compliance with relevant regulations and laws. The researcher will not share the subject's information with others without permission. The results of this trial may be published at conferences or journals in the future, but the research team assures that the subjects' personal information will not be disclosed.

## 3. Discussion

Movement dysfunction of the upper limb after stroke is mainly characterized by dystonia and abnormal movement patterns, resulting in difficulty in regulating the patient's movements and inability to perform fine movements, which has a serious impact on the performance of various functional tasks in daily life (41, 42). In addition, stroke patients often present with superficial sensory and proprioceptive abnormalities. Sensory function is essential for grasping movements and skilled object manipulation, and the reduction of sensory feedback weakens fine control of objects (43). Derakhshanfar et al. (44) pointed out that sensory stimulation is an effective way to improve motor function and ADL in patients.

Yan (45) proposed “combination of the effective approaches between the brain and the limbs” for the treatment of functional impairment in stroke patients in recent years. This technology involves the application of brain and limb rehabilitation techniques simultaneously or in a certain treatment sequence to patients with brain injury, thus activating the central-peripheral regulatory function and bringing about a synergistic effect of the two techniques. In addition, sensorimotor integration also plays an important role in the pathophysiological mechanisms of poststroke dyskinesia, which is the process by which input sensory signals assist in the execution of motor programs after acting through the integration of the central nervous system (46). It has been found that sensorimotor integration is impaired in patients with stroke motor disorders and the degree of impairment is positively correlated with the severity of motor function (47). Therefore, peripheral and central sensory information stimulation contributes to the improvement of movement function after stroke, and it has important clinical significance in the treatment of patients from the aspect of sensorimotor integration.

Tang et al. (48) concluded that simultaneous head-acupuncture rehabilitation therapy can stimulate peripheral sensation, increase cortical activity, and improve motor function in stroke patients (48). The ASRT adopted in this study is a technique in which acupuncture is accompanied by simultaneous rehabilitation training. The therapy adopts a combination of central intervention and peripheral intervention, with simultaneous brain stimulation and limb stimulation, forming a “closed-loop” information feedback (49), which can maximize the integration of sensory-motor signals and strengthen sensory stimulation, helping to enhance the control of motor function by the nervous system and prompting the central nervous system to remodel and form new functional areas after brain injury, thus improving patient function.

This study will compare the safety and efficacy of several different “simultaneous treatment modalities” of acupuncture and rehabilitation and will be evaluated using a combination of subjective and objective indicators such as FMA-UE, SEP, UEFT, WMFT, reNSA, DTI, and MBI to investigate whether there is a synergistic effect between scalp acupuncture and intradermal acupuncture, which will undoubtedly help to determine a better treatment option. We hope to select a more effective and safer therapy to improve upper extremity function in stroke patients.

This study protocol cannot be blinded to patients and therapists and physicians and is limited by the single-center design. In addition, the follow-up period is short (6 months). These limitations may affect the reliability of the results. However, we remain to hope that the results of this RCT will provide high-quality clinical evidence on whether ASRT improves motor and sensory function of the upper limb, and ADL in patients with stroke, and determine whether there is a synergistic effect between scalp acupuncture and intradermal acupuncture. If the results of this trial show that this treatment option improves efficacy and has a good safety profile, our research team will conduct a multi-center, larger sample RCT to further determine its efficacy and safety.

## 4. Trial status

Patient recruitment for this trial began on January 1, 2023 and is expected to be completed by December 31, 2023, with data analysis to be completed by January 31, 2024.

## Ethics statement

This study protocol was approved by the Ethics Committee of the Affiliated Hospital of Shandong University of Traditional Chinese Medicine (approval no. 2022-080-KY) on August 5, 2022.

## Author contributions

XLi designed the trial and was responsible for the manuscript. ZY and XY drafted the manuscript together. FQ and TM were responsible for data collection. XLe performed the recruitment. ZY and JZ were responsible for the statistical design of this study. HB contributed to the revision of the study protocol. All authors contributed to the article and approved the submitted version.
